# The Role of Beauty Salons in Community Health Promotion: Exploring Health Engagement and Social Connectivity in Japanese Hair Salons

**DOI:** 10.7759/cureus.79511

**Published:** 2025-02-23

**Authors:** Yuta Horinishi, Rimi Horinishi, Chiaki Sano, Ryuichi Ohta

**Affiliations:** 1 Community Care, Unnan City Hospital, Unnan, JPN; 2 Family Medicine, Unnan City Hospital, Unnan, JPN; 3 Community Medicine Management, Faculty of Medicine, Shimane University, Izumo, JPN

**Keywords:** beauty culture, beauty salon, community health services, general medicine, health promotion, japan, patient engagement, rural, social support

## Abstract

Introduction

Beauty salons serve as more than just spaces for personal grooming; they are social hubs that facilitate interpersonal connections. Given the increasing importance of social engagement in public health, beauty salons may have a role in promoting health awareness and reducing social isolation. While international studies have demonstrated the effectiveness of salon-based health interventions, there is limited research on the role of Japanese beauty salons in community health promotion. This study aims to explore health-related conversations and awareness among hairstylists in Japan.

Method

A survey-based study was conducted at Hair Lab En Luce, a beauty salon in Matsue City, Japan. Five hairstylists and assistants recorded customer interactions over one month in June 2022. The survey collected data on customer demographics, conversation topics, hairstylists’ emotional responses, and perceived actions for future engagement. Thematic analysis was employed to identify key themes related to health engagement.

Results

A total of 127 survey responses were analyzed. Health-related topics constituted 27.6% of conversations, demonstrating hairstylists’ engagement in customers’ well-being. Thematic analysis revealed three key themes: continuity with customers, trust-based customer-hairstylist relationships, and the expanding role of beauty salons in health promotion. Hairstylists often provided informal health advice and observed scalp and skin conditions, occasionally referring customers to healthcare professionals.

Conclusion

Japanese beauty salons function as spaces for both personal care and social support. Strengthening collaborations between hairstylists and healthcare providers may enhance their role in community health promotion. Future research should assess the effectiveness of salon-based health interventions in Japan.

## Introduction

Beauty salons provide grooming services and significant community spaces that foster and maintain social connections [1]. In modern society, social isolation and weakening interpersonal relationships have been shown to impact mental and physical health [2]. Addressing these issues requires increasing opportunities for interpersonal interaction and strengthening social relationships [3]. Beauty salons serve as environments where diverse individuals regularly visit and engage in natural conversations, suggesting their potential role in mitigating isolation and facilitating social network formation [4].

The role of hairstylists extends beyond providing hair care, as they can engage with customers on health-related matters through their ongoing relationships. Previous studies have reported that many hairstylists are willing to provide health information to their clients and, in practice, offer advice on nutrition, exercise, and chronic disease prevention [5]. In some countries, educational programs have been implemented to train hairstylists in raising awareness of lifestyle-related disease prevention and cancer screenings, enabling them to contribute to health consciousness through their conversations with customers [6,7]. Such interventions suggest that hairstylists can be key figures in promoting community health [6,7].

However, the roles and functions of beauty salons within local communities will likely differ significantly depending on the country and cultural context. For instance, community-based interventions utilizing beauty salons to promote hypertension and prostate cancer screenings have been implemented in the United States, demonstrating their effectiveness [8]. In contrast, in Japan, there is a lack of research on how hairstylists engage with clients' health and how they can serve as agents of health promotion.

Given society's increasing diversity, elucidating the health-supporting functions of beauty salons may enable their utilization as a novel community resource for improving public health. Therefore, this study explores the current state and potential role of beauty salons in health engagement in Japan by surveying conversations in beauty salons and hairstylists' health awareness, followed by thematic analysis.

## Materials and methods

Study setting

This study was conducted at Hair Lab En Luce, a beauty salon in Tawarayama, Matsue City, Shimane Prefecture, Japan. The salon has three hairstylists and two assistants and is open 24 days a month. The average number of customers per month is approximately 250.

Shimane Prefecture is part of the Chūgoku region in Japan, with its capital city being Matsue. It is located in the western part of Honshu and occupies the west side of the San’in region. As of December 1, 2024, Matsue City had a population of 197,102, an area of 572.96 km², and a population density of 344 people/km².

According to the 2022 Hygiene Administration Report, there were 269,889 beauty salons across Japan, with 216.0 salons per 100,000 people. In contrast, Matsue City had 558 beauty salons, equating to 278.0 salons per 100,000 people, exceeding the national average.

Participants

The participants included three hairstylists and two assistants working at Hair Lab En Luce in Tawarayama, Matsue City, and customers visiting the salon.

Data collection

A survey was conducted over one month in June 2022, targeting beauty salon staff to collect information on customer conversations. The survey questionnaire was developed based on a preliminary literature review on customer-staff interactions in service industries and consultations with communication studies and psychology experts. A pilot test was conducted with a small group of beauty salon staff to assess the questions' clarity, relevance, and comprehensiveness. Based on the feedback, minor modifications were made to improve wording and ensure ease of understanding. The finalized survey included the following items: customer’s age, customer’s gender, content of the conversation with the customer, staff's emotions during the conversation, and actions the staff felt that they could take in the future (Appendix 1). For conversation content, respondents were asked to select from predefined categories, including family, food and nutrition, health and medical topics, work, personal life, hair and beauty, hobbies, and current events.

Additionally, an open-ended section was provided for further description. Items 4 and 5 were collected using an open-ended response format. To ensure content validity, the questionnaire was reviewed by professionals in qualitative research and salon management before distribution. Before the study, written explanations were provided to Hair Lab En Luce staff and customers, and informed consent was obtained.

Data analysis: thematic analysis

Thematic analysis was conducted following six steps: (1) familiarization with the data, (2) generating initial codes, (3) searching for themes, (4) reviewing themes, (5) defining and naming themes, and (6) producing the final report [9]. The first and second authors thoroughly reviewed the survey results. The first author performed the initial content coding and iteratively refined the codebook. The second author independently coded the data. Both authors then discussed the coding consistency based on the codebook and extracted conceptual categories by comparing the research materials with the assigned codes. Major themes were identified from the extracted concepts. Discussions continued until mutual agreement was reached, ensuring that all possible concepts and themes were covered, leading to theoretical saturation. Finally, all authors discussed the themes and concepts and reached a consensus.

Ethical considerations

This study was approved by the Clinical Ethics Committee of Unnan City Hospital (Approval Number: 20220005).

## Results

Survey responses regarding their conversations with customers were collected from five staff members at Hair Lab En Luce. All participating staff members were female. A total of 127 survey responses were obtained, with the gender distribution of customers being 18 males and 109 females.

The content of the conversations was categorized as follows: family: 32/127 (25.2%), food and nutrition: 6/127 (4.7%), health and medical topics: 35/127 (27.6%), work: 11/127 (8.7%), personal life: 35/127 (27.6%), hair and beauty: 4/127 (3.1%), hobbies: 2/127 (1.6%), and current events: 2/127 (1.6%).

Thematic analysis regarding hair salons’ role in health promotion in customer relationships

Three key themes emerged through thematic analysis: continuity with customers, the relationship between customers and hairstylists, and the expansion of beauty salons’ roles. Continuity with customers reflects how hairstylists engage in customers’ life events over time, fostering deeper connections that often extend to entire families as they progress through different life stages. The relationship between customers and hairstylists is built on trust as hairstylists strive to achieve desired aesthetic outcomes. This trust extends across generations, strengthening multigenerational bonds. Additionally, these customer interactions often motivate hairstylists to improve their expertise. The expansion of beauty salons’ roles highlights their growing involvement in health-related discussions. Hairstylists observe scalp and skin conditions, sometimes collaborating with healthcare professionals. Salons also serve as informal health information sources and personalized spaces promoting customer well-being beyond hair care (Table 1).

**Table 1:** Themes and concepts regarding the role of hair salons in health promotion in customer relationships

Theme	Concept
Continuity with Customers	Listening to life changes through engagement in life events
	Increased involvement with the entire family due to changes in the life cycle
Relationship Between Customers and Hairstylists	Building trust through the pursuit of the customer’s desired outcome
	Expansion of multigenerational relationships through temporal continuity
	Hairstylists’ motivation for learning through customer interactions
Expansion of the Role of Beauty Salons	Developing collaboration with healthcare professionals through discussions on scalp and skin conditions
	The role of beauty salons as a source of health information
	Beauty salons as comfortable and personalized spaces

**Figure 1:**
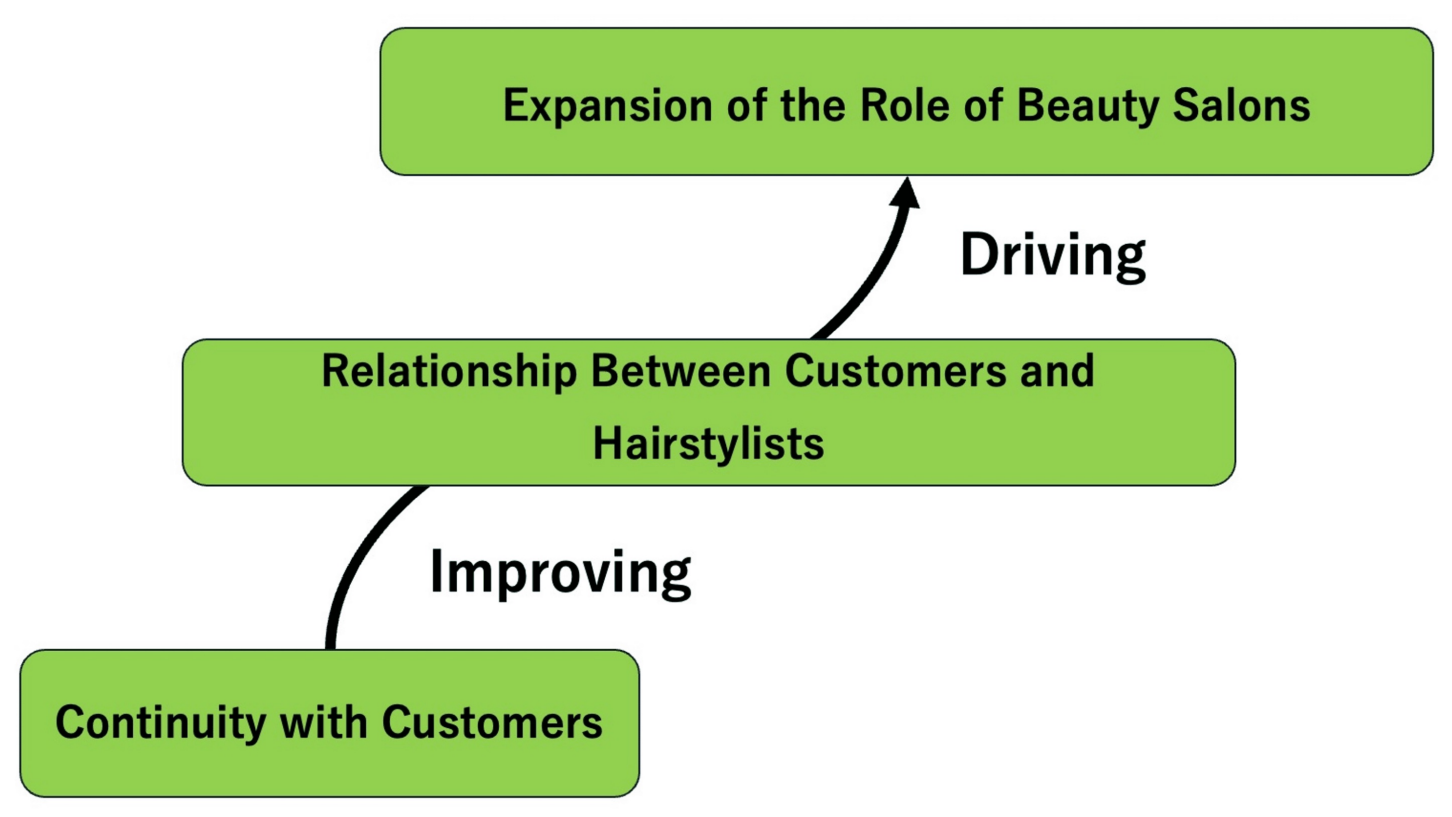
Conceptual figure regarding the role of hair salons in health promotion in customer relationships Image credit: Ryuichi Ohta

Figure 1 shows the conceptual figure.

Continuity with customers

Listening to Life Changes Through Engagement in Life Events

A beauty salon is not merely a place for hair maintenance but also a space where customers share their emotions and build supportive relationships as they navigate various life events. Customers who regularly visit salons for hair maintenance also engage with their hairstylists during significant milestones, such as entrance ceremonies, graduations, and weddings. Hairstylists contribute to customers' emotional well-being by providing the most suitable hairstyle for each occasion while offering emotional support.

One example involved a female customer in her 30s preparing for her wedding. While she was busy with preparations, she also felt excitement and anticipation. Her hairstylist engaged in discussions about her preferred hairstyle and was scheduled to handle her wedding day hair styling. Through this interaction, the hairstylist expressed gratitude for building such a relationship and felt fulfilled in her work. For the customer, the hairstylist was a service provider and a trusted companion who shared her essential life moments.

Similarly, a teenage female customer, preparing for her graduation ceremony, had recently experienced a breakup and was feeling emotional distress. Despite this, she expressed a desire to look her best for the ceremony. She confided in her hairstylist, who empathized with her emotions and acknowledged her pain by saying, “That must be tough.” At the same time, the hairstylist helped her consider a hairstyle that would boost her confidence for the ceremony.

Such interactions extended beyond mere beauty services, providing psychological support and helping customers mentally prepare for significant life events.

Increased Involvement with the Entire Family Due to Changes in the Life Cycle

As long-term customers progress through different life stages, beauty salons begin to play a role not only for individuals but also as an integral part of their families. Customers who become mothers start bringing their children to the salon for their Shichi-Go-Sanceremonies or their first haircut, and they visit more frequently to prepare for family events. Through these interactions, beauty salons become deeply involved not only in the personal growth of customers but also in the transformations within their families.

One example involved a male teenage customer who had been visiting the salon since infancy. Now a high school student, he expressed concern about his hair texture becoming unmanageable during the rainy season. The hairstylist, who had been cutting his hair since childhood, noticed his growing attention to personal style and responded, “Now that you're in high school, your style becomes more important. I’ll make sure to honor your preferences.” This long-term relationship allowed the hairstylist to adapt to the customer’s evolving needs while maintaining a strong foundation of trust.

Similarly, a woman in her 30s visited the salon after attending her child’s first sports day. She shared, “It was tough preparing from early in the morning, but I had such a great time.” The hairstylist, who had also recently experienced a sports day event, responded, “Ours was on the same day! It’s exhausting. But seeing the kids trying their best makes it all worth it.” These shared experiences and mutual empathy strengthened the bond between the hairstylist and the customer, fostering a more profound relationship built on trust and shared life events.

Customer-hairstylist relationship

Building Trust Through the Pursuit of the Customer’s Desired Outcome

Hairstylists are not merely providers of haircuts and styling but also dedicated partners in helping customers achieve their desired hair color and skincare routines. Hairstylists play a crucial role in establishing trust-based relationships by actively engaging with customers, understanding their preferences, and working collaboratively to meet their expectations.

One day, a woman in her 20s visited the salon and requested, “I want my hair to be a beautiful shade of purple.” Having chosen purple before, she was greeted by her hairstylist's warm response: “Purple again this time! I’ll do my best to ensure it turns out just how you like it.” Rather than simply applying the requested color, the hairstylist considered the customer's preferences and past styles, demonstrating a commitment to personalized service. This attentiveness contributed to the customer’s satisfaction and trust in the hairstylist.

Many customers seek concrete solutions for concerns related to their hair and skin. Hairstylists, leveraging their expertise, provide tailored advice based on the customer's age and lifestyle, enhancing their sense of security and confidence in self-care. For instance, a man in his 40s asked for guidance on wrinkle care suitable for his age. The hairstylist responded, “Let me show you how to use moisturizer and lotion properly. Additionally, sun protection is crucial for preventing dark spots.” By offering professional knowledge alongside practical, easy-to-implement care methods, hairstylists help customers feel assured that they can adopt a routine that suits their needs.

Expansion of Multigenerational Relationships Through Temporal Continuity

The relationships between hairstylists and customers of the same generation often resembled friendships or the camaraderie of fellow parents. They shared various life concerns, supported each other, and sought to grow together. For hairstylists, their role extended beyond simply maintaining hair; they became companions in learning and mutual encouragement.

For instance, when a woman in her 20s confided in her hairstylist, saying, “There are times when I lose control of my emotions and end up troubling those around me,” the hairstylist responded with empathy, “Let’s reflect together on when it becomes difficult to control your emotions and think about possible coping strategies.” This interaction highlighted how hairstylists were not merely service providers but also life counselors, offering emotional support. The beauty salon was not just a place for hair care but also a space where physical appearance and emotional well-being were nurtured.

For long-time older customers, hairstylists often took on a role akin to that of a daughter or granddaughter. Each visit to the salon fostered a warm, familial relationship in which the hairstylist genuinely cared for the customer’s well-being. One day, a woman in her 70s visited the salon and asked, “The last time I was here, you seemed a bit down. Are you feeling better now?” demonstrating concern for the hairstylist. The hairstylist replied, “You always listen to my worries, and I’ve learned so much from your different perspectives and values - it’s helped me grow.”

This exchange illustrated that the relationship was not one-sided; hairstylists support their customers and learn from them, fostering their personal growth.

Hairstylists’ Motivation for Learning Through Customer Interactions

Through daily conversations with numerous customers, hairstylists deepen their knowledge of hair care and beauty and gain insights that contribute to their personal growth. Interactions with customers of the same generation or those who approach life positively serve as a significant source of motivation. Beauty salons are not merely places for hair maintenance but spaces where people share their daily concerns and experiences.

For instance, when a female customer in her 30s shared, “Maintaining relationships with other moms in the neighborhood is exhausting, but I’m doing my best,” the hairstylist responded, “As children grow, social interactions expand, and it can be challenging. But listening to your experiences has taught me different ways of engaging with others, and it’s been motivating for me.” This exchange illustrates how hairstylists learn about the diversity of human relationships and refine their interpersonal skills through customer interactions. By empathizing with their customers and sometimes gaining new perspectives, hairstylists enhance their approach to customer service and develop their outlook on life.

Hairstylists, too, find encouragement in their customers' words. For example, when a hairstylist preparing for maternity leave was told by a woman in her 20s, “You must be excited about your baby! I wish you all the best for the delivery,” she felt deeply grateful and responded, “I’ve received so many encouraging words before my maternity leave - I'm truly thankful! I’ll do my best to have a healthy delivery and return to work with renewed energy!”

These interactions are not one-sided; their customers often support and uplift hairstylists. The stronger their relationships with customers become, the more these exchanges serve as a source of encouragement and motivation, fueling their passion for their work and personal lives.

Expansion of the role of beauty salons

Developing Collaboration with Healthcare Professionals Through Discussions on Scalp and Skin Conditions

Beauty salons serve as places for hair and skin care and spaces where customers can discuss daily concerns and health-related issues. Hairstylists often receive consultations on a wide range of topics, including skin troubles, hair loss due to chemotherapy, chronic health conditions, and even difficulties within the home. By connecting customers with appropriate professionals, hairstylists facilitate collaboration with healthcare providers and community support services, ensuring that customers receive the necessary assistance.

For example, when a woman in her 70s undergoing chemotherapy for breast cancer expressed concerns about hair loss, her hairstylist did more than offer hair care advice. She listened attentively to the customer's worries and provided suggestions on wig options and skincare routines. The hairstylist reassured her, saying, “It would be great if you have a primary care doctor to talk to easily. I can also ask my husband, who is a physician, for advice.” This interaction highlights the hairstylist’s role in acknowledging delicate concerns such as hair loss and skin changes while serving as a bridge to medical professionals. Many customers find it easier to discuss such issues in a beauty salon's relaxed atmosphere than in a hospital setting, and these conversations often lead to seeking medical attention when necessary.

Similarly, when a woman in her 30s mentioned, “I’m struggling to manage my hypothyroidism. The fatigue and swelling won’t improve, and I’m worried,” her hairstylist responded, “My husband is a doctor - I can ask him about it!” This interaction demonstrates that hairstylists are hair and skincare advisors and key figures in facilitating discussions about health concerns and connecting customers with specialists. Since thyroid dysfunction often manifests in physical changes such as swelling and hair texture alterations, hairstylists may be among the first to notice these symptoms. By leveraging their observational skills and networks, they contribute to customers’ overall health management by guiding them toward appropriate medical care.

Beauty salons become a rare source of comfort for some individuals living with illness. One woman in her 80s shared, “Ever since my pancreaticoduodenectomy, I’ve been experiencing recurrent cholangitis, but coming to the salon is one of the few pleasures in my life.” The hairstylist, recognizing her as someone her husband (a physician) had treated during medical school, responded, “He hasn’t seen you in a while, but I’m sure he’d be happy to hear how you’re doing.”

These examples illustrate that beauty salons are more than just places for aesthetic care; they serve as emotional sanctuaries for customers and, through their connections with healthcare professionals, as venues for establishing better support systems.

The Role of Beauty Salons as a Source of Health Information

Beauty salons function not only as places for hair and skin care but also as spaces where customers can regularly obtain health-related information. Since hairstylists possess expertise in hair and skincare while taking an interest in nutrition and physical well-being, customers feel comfortable discussing their health concerns with them. Through casual conversations during daily treatments, hairstylists naturally provide hints and advice for maintaining good health, positioning beauty salons as accessible sources of health information.

For example, when a woman in her 60s mentioned, “I have chronic back pain, but hanging from a horizontal bar relieves it,” the hairstylist went beyond simply empathizing and provided advice on stretching and massage techniques. By understanding customers' physical conditions and suggesting appropriate self-care methods, hairstylists support them in adopting sustainable health habits that can be easily integrated into daily life. These interactions allow customers to learn simple self-care techniques before seeking medical attention, ultimately contributing to better health management.

Similarly, when a man in his 50s asked, “Do you have any suggestions for low-calorie snacks?” the hairstylist recommended, “How about lightly grilled Atsuage (thick fried tofu) with bonito flakes and ponzu sauce?” This example highlights how hairstylists acquire knowledge about diet and nutrition, enabling them to propose meal ideas that align with customers’ lifestyles and health goals.

As health-conscious customers grow, providing information on “sustainable and healthy eating habits” has become an increasingly valuable service within beauty salons.

Beauty Salons as Comfortable and Personalized Spaces

Beauty salons serve as places for hair care and as “special spaces” where customers feel safe sharing their daily concerns and emotions. Unlike the home (a first place) or the workplace and community (a second place), beauty salons provide a unique environment with a different level of personal distance, allowing customers to open up more quickly. Within this space, hairstylists build trust with their clients by listening attentively, empathizing, and offering emotional support. As a result, customers find it natural to discuss hair-related concerns and personal and interpersonal challenges.

For example, a woman in her 70s shared, “I always have such a great time here because you listen to me. I don’t even consider going to another salon.” In response, her hairstylist expressed appreciation, saying, “Thank you for sharing your hair concerns and thoughts with me. I’m truly grateful for your kind words.”

This interaction highlights the role of beauty salons as more than just places for aesthetic treatments - they also function as spaces for emotional support. By genuinely listening to customers and acknowledging the worries they carry in their daily lives, hairstylists create a welcoming and accepting environment. As a result, beauty salons become “a special place where customers feel understood and valued.”

## Discussion

This study conducted a thematic analysis of conversations between customers and hairstylists in beauty salons to explore awareness of health needs and examine the potential role of beauty salons in community health promotion. The findings suggest that beauty salons serve as spaces for beauty treatments and potential health hubs within local communities. Key themes identified include the continuity of relationships between customers and hairstylists, the expanding social role of beauty salons, and the provision of health-related information. These results indicate that beauty salons can contribute to community well-being by functioning as more than just aesthetic service providers, offering health awareness and support opportunities.

Beauty salons vary significantly across different countries and cultures, and Japanese beauty salons are characterized by “continuity in dialogue” and a “relaxing environment.” This study found that relationships between customers and hairstylists tend to be long-term, allowing hairstylists to accompany customers through significant life events, such as marriage, childbirth, school progression, and employment. These findings suggest that Japanese beauty salons function as places for beauty treatments and as “spaces where personal relationships are cultivated" [10]. Additionally, Japanese beauty salons may function as “safe spaces for consultation.” Beyond providing expert advice on hair and skin care, they also naturally facilitate exchanges of information on health and lifestyle [11]. Previous international studies have demonstrated the effectiveness of health promotion programs based in barbershops and beauty salons [12]. In rural contexts, as people can lose the opportunity to have dialogues about their health conditions, salons, including beauty salons and community meetings, should be promoted [13].

However, unlike beauty salons in the United States and Europe, Japanese beauty salons have yet to actively function as venues for health interventions. In the United States, initiatives such as Kaiser Permanente’s Good Health & Great Hair Program have been implemented to use beauty salons to disseminate health information and encourage community members to seek medical care [14]. Japanese beauty salons may also have the potential to strengthen their role as hubs for health promotion. To promote the health benefits of Japanese beauty salons, more research should investigate the effectiveness of Japanese beauty salons on users’ health conditions [15].

This study found that conversations related to health accounted for 27.6% of all interactions, indicating that hairstylists show interest in customers' health concerns and, in some cases, provide information or refer them to appropriate professionals. Similar trends have been reported in international research [6]. For instance, some studies suggest that beauty salons can help reduce social isolation and improve mental well-being, particularly for older adults and individuals with chronic illnesses [16]. Another notable characteristic of Japanese beauty salons is the long-term relationships built between hairstylists and customers, often extending across generations [17]. This study identified cases where three generations of a family visited the same salon, forming intergenerational connections within the community. This reflects the nature of Japanese local communities and suggests that beauty salons play a role in strengthening family and regional ties [18].

This study identified cases in which hairstylists detected health concerns through customers' scalp and skin conditions and, when necessary, referred them to healthcare professionals [19]. Health intervention programs in beauty salons have been actively implemented in the United States. A systematic review by Palmer et al. demonstrated that health education programs conducted through beauty salons significantly improved health outcomes, particularly among African American communities [6]. Additionally, hairstylists have been reported to play a role in promoting cancer screenings. For example, the Hair Salon Stylists as Breast Cancer Prevention Lay Health Advisors study suggested that hairstylists could provide breast cancer prevention information to customers and potentially increase screening rates [20].

Although Japanese beauty salons also have the potential to serve as hubs for health information dissemination, as this study shows, their role in this capacity has not yet been established. Medical professionals, including general physicians, can promote their role in health promotion as general physicians manage various patients in medical institutions and communities [21,22]. Especially in rural Japanese contexts, healthcare resources can be limited so that a beauty salon can be a health resource [23]. This article shows that hairstylists are expected to enhance their health literacy and connect customers with appropriate healthcare professionals. Strengthening collaboration with primary care physicians in Japanese communities could lead to more comprehensive health support.

This study has several limitations. First, it was conducted at a single beauty salon in Matsue City, Shimane Prefecture, making it difficult to generalize the findings to other regions or beauty salons with different cultural backgrounds. Additionally, the sample size was limited (five hairstylists and 127 customers), raising concerns regarding the results' reliability and reproducibility. Furthermore, since the study relied solely on survey data, it did not account for non-verbal elements such as gestures or tone of voice, which may have provided additional insights. Future research should conduct large-scale studies involving beauty salons in diverse regions and cultural contexts to assess the effectiveness of health interventions facilitated by hairstylists. Moreover, incorporating observational studies and interviews would allow for a deeper qualitative analysis of the hairstylist-customer relationship, further elucidating the role of beauty salons in community health promotion.

## Conclusions

This study suggests that hairstylists demonstrate a strong interest in health and actively provide health-related information to their customers regardless of cultural differences. Beauty salons can function as spaces for hair care, consultation venues, and hubs connecting local communities. In particular, the continuity of relationships between hairstylists and customers facilitates health information dissemination and strengthens social connections. Future research should explore the potential of utilizing beauty salons as platforms for community health initiatives.

## Appendices

Appendix 1. Customer conversation survey for beauty salon staff

Instructions

Thank you for participating in this survey. The purpose of this survey is to understand the nature of conversations between beauty salon staff and customers. Please answer the following questions based on your experience. Your responses will remain confidential.

1. Customer Information

Age: ☐ Under 20 ☐ 20-29 ☐ 30-39 ☐ 40-49 ☐ 50-59 ☐ 60 or older

Gender: ☐ Male ☐ Female ☐ Other

2. Conversation Content

Please select all topics that were discussed during the conversation with the customer: ☐ Family ☐ Food and Nutrition ☐ Health and Medical Topics ☐ Work ☐ Personal Life ☐ Hair and Beauty ☐ Hobbies ☐ Current Events ☐ Other (Please specify): ___________________________

3. Staff’s Emotions During the Conversation

How did you feel during the conversation? Please describe your emotions in your own words.

4. Actions You Felt You Could Take in the Future

Based on this conversation, did you feel there were any actions you could take to improve customer service or communication in the future? Please describe.

5. Additional Comments (Optional)

If you have any other thoughts or experiences related to customer conversations, please write them here.

Consent Statement

By completing this survey, you agree to participate in this study. Your responses will remain anonymous and will be used for research purposes only.
